# Induction of ROS Overload by Alantolactone Prompts Oxidative DNA Damage and Apoptosis in Colorectal Cancer Cells

**DOI:** 10.3390/ijms17040558

**Published:** 2016-04-14

**Authors:** Yushuang Ding, Hongge Wang, Jiajing Niu, Manyu Luo, Yangmei Gou, Lining Miao, Zhihua Zou, Ying Cheng

**Affiliations:** 1 Department of Radiotherapy, the Second Hospital of Jilin University, 218 Ziqiang Street, Changchun 130041, China; dingyushuang000@sina.com; 2 School of Life Sciences, Jilin University, 2699 Qianjin Street, Changchun 130012, China; wanghg13@mails.jlu.edu.cn (H.W.); niujj14@mails.jlu.edu.cn (J.N.); yuzixin093@163.com (Y.G.); 3 Department of Nephrology, the Second Hospital of Jilin University, 218 Ziqiang Street, Changchun 130041, China; robertlmy@sina.com (M.L.); miaolining55@163.com (L.M.); 4 Department of Thoracic Oncology, Jilin Provincial Cancer Hospital, 1018 Huguang Street, Changchun 130012, China

**Keywords:** alantolactone, DNA damage, 8-oxoG, apoptosis, cell cycle arrest, colorectal cancer

## Abstract

Cancer cells typically display higher than normal levels of reactive oxygen species (ROS), which may promote cancer development and progression but may also render the cancer cells more vulnerable to further ROS insult. Indeed, many of the current anticancer therapeutics kill cancer cells via induction of oxidative stress, though they target both cancer and normal cells. Recently, alantolactone (ATL), a natural sesquiterpene lactone, has been shown to induce apoptosis by increasing ROS levels specifically in cancer cells; however, the molecular mechanisms linking ROS overproduction to apoptosis remain unclear. Here we show that the ATL-induced ROS overload in human SW480 and SW1116 colorectal cancer cells was followed by a prominent accumulation of cellular oxidized guanine (8-oxoG) and immediate increase in the number of DNA strand breaks, indicating that increased ROS resulted in extensive oxidative DNA damage. Consequently, the G_1_/S-CDK suppresser CDKN1B (p21) and pro-apoptotic proteins Bax and activated caspase-3 were upregulated, while anti-apoptotic Bcl-2 was downregulated, which were followed by cell cycle arrest at G_1_ and marked apoptosis in ATL-treated cancer but not non-cancer cells. These results suggest that the ATL-induced ROS overload triggers cell death through induction of massive oxidative DNA damage and subsequent activation of the intrinsic apoptosis pathway.

## 1. Introduction

Given the severe side-effects and unsatisfactory outcomes associated with conventional anticancer therapeutics, it is important to find novel anticancer strategies that can more specifically and effectively eradicate all cancerous cells. Targeting cancer-specific genetic and biochemical hallmarks that are crucial for the survival of cancer cells but dispensable for normal cells would be clinically beneficial. Research in the past has focused heavily on genetic targets such as BCR-ABL, B-RAF, ALK, PARP, Chk1, and ATR, which has resulted in a few impressive small molecule and antibody drugs [1]. However, such a gene-specific approach is limited by the diversity of cancer genotypes, intra-tumor heterogeneity and lack of actionable targets in some cancers [2]. Furthermore, drug resistance is still a general problem when targeting specific genetic mutations due to the genomic instability and consequent genetic adaptability of cancer cells [3]. In this regard, targeting more generic biochemical or phenotypic vulnerabilities of cancer cells appears to be more attractive as it attacks shared fragilities of cancer cells irrespective of the cancer genotype. Such an approach can be applied more broadly and is less likely to suffer from drug resistance [4,5]. Cancer cells rewire their metabolism to survive and to promote proliferation, growth, and metastasis [6,7]. Knowledge about cancer-sustaining metabolic pathways is revealing many critical phenotypic biochemical differences between normal and cancer cells, which are valuable for developing more effective and specific anticancer agents [8,9,10].

Due to altered metabolism, dysfunctional mitochondria, hypoxia, and oncogenic signaling, cancer cells typically exhibit high levels of reactive oxygen species (ROS) [11,12,13]. Elevated ROS may promote tumor onset and progression by increasing DNA damage and genome instability, as well as inducing pro-tumorigenic signaling [12]. At the same time, extensive oxidative DNA damage caused by elevated ROS may abrogate the cellular repair capacity to trigger DNA damage response (DDR), which then lead to cell cycle arrest, premature cellular senescence, or programmed cell death, therefore blocking cancer development and progression [14]. In addition, ROS-induced oxidative stress can also directly provoke programmed cell death including apoptosis, autophagy, necroptosis, and ferroptosis [15,16]. Large-scale clinical trials have shown that suppressing ROS by antioxidants had no effects on cancer progression, and some trials showed that antioxidants increased cancer risks [17,18,19]. Furthermore, recent studies found that antioxidants accelerated the progression of lung cancer [20] and increased melanoma metastasis in mice [21], while oxidative stress inhibited distant metastasis by human melanoma cells [22]. Notably, induction of Nrf2, a master transcriptional activator of antioxidant proteins, promoted tumorigenesis [23]. Thus, despite its potential mutagenic activity, ROS most likely play very important roles in suppressing cancer development and progression. Supporting this idea, a vast number of studies have found that most cancer cells exhibit significantly enhanced anti-oxidative systems and are critically dependent on antioxidants, nucleotide pool sanitizing enzymes, and DNA repair processes for survival [24]. For example, suppressing the activity of the nucleotide pool sanitizing enzyme MTH1 effectively killed cancer cells both *in vitro* and *in vivo* through induction of widespread oxidative DNA damage [25,26,27]. Thus, targeting the antioxidant capacity or further promoting oxidative stress in cancer cells can be exploited as selective anticancer strategies.

Conventional radiotherapy and many chemotherapeutic drugs rely on induction of ROS overproduction to kill cancer cells. However, these treatments target cancer and normal cells indiscriminately. Recently, a variety of chemical compounds, mostly natural products, have been identified that induce programmed cell death specifically in cancer cells by promoting ROS overload [28,29,30,31,32,33,34]. One of these compounds, alantolactone (ATL), is a natural sesquiterpene lactone and the major pharmacological ingredient of the medicinal plant *Inula helenium* [35]. It has previously been shown to have anti-inflammatory and anti-microbial bioactivity, with no significant toxicity against normal cells. Some recent studies showed that ATL promoted ROS accumulation specifically in cancer cells [36,37,38,39], through depletion of glutathione (GSH) [36,37] or inhibition of thioredoxin reductase (TrxR) [38]. The ROS overload induced by ATL was followed by apoptotic cell death, which was blocked by the specific ROS inhibitor, *N*-Acetyl Cysteine (NAC), indicating that apoptosis was driven by the elevated ROS [36,37,38,39]. However, the molecular pathways linking ATL-induced ROS overproduction to cancer cell apoptosis have remained unclear. Given that single-agent anticancer therapy frequently encounters drug resistance, it is important to design therapies using combinations of drugs that act synergistically. To this end, thorough understanding of the mechanisms underlying the activities of individual drugs is necessary. Here we show that treatment of human SW480 and SW1116 colorectal cancer cells with ATL caused a prominent increase in ROS levels, which was followed by extensive oxidative DNA damage and subsequent cell cycle arrest and apoptosis. Inhibition of ROS by NAC prevented the DNA damage and cancer cell death, suggesting that the ATL-induced ROS overload inhibits cancer cell survival through induction of substantial oxidative DNA damage and activation of DDR signaling. Understanding the details behind the cancer-specific cytotoxicity of ATL will help in the design of synergistic anticancer drug combinations using ATL-like compounds.

## 2. Results and Discussion

### 2.1. Cytotoxicity of Alantolactone (ATL) against SW480 and SW1116 Colorectal Cancer Cells

**Figure 1:**
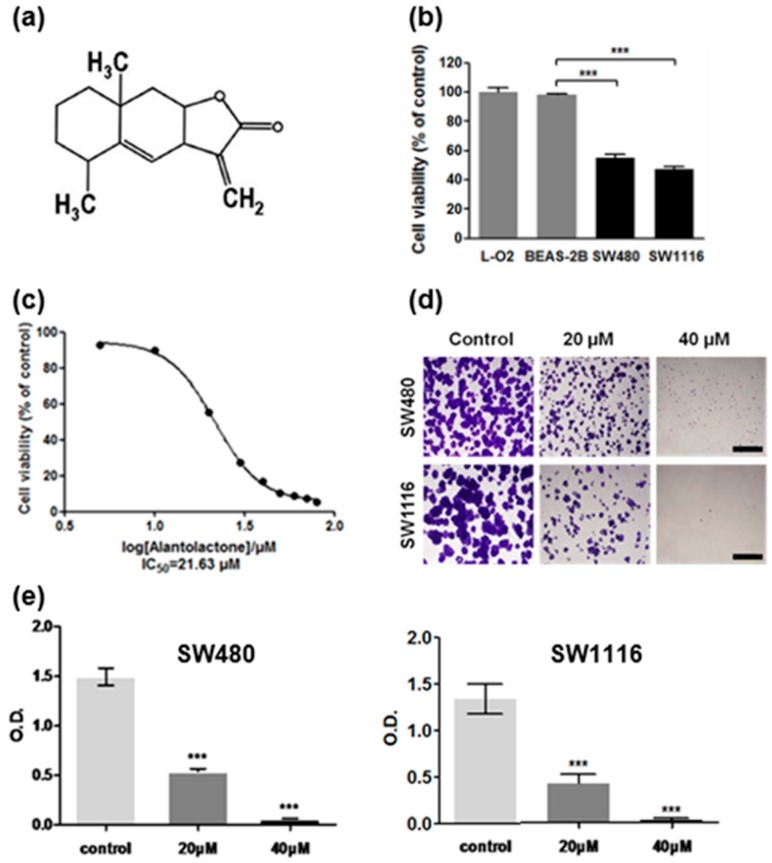
Alantolactone (ATL) potently suppressed the growth of the SW480 and SW1116 colorectal cancer cells. (**a**) Chemical structure of ATL; (**b**) ATL cytotoxicity determined by the MTT assay: treatment by 20 μM ATL for 24 h potently suppressed the growth of SW480 and SW1116 colorectal cancer but not the non-cancer BEAS-2B and L-O2 cells (each treatment was done in triplicate, *** *p <* 0.001 *vs.* BEAS-2B); (**c**) Detailed dose-response studies: 24 h treatment by ATL inhibited the proliferation of SW480 and SW1116 cancer cells with an *IC*_50_ of 21.63 and 18.14 μM, respectively; (**d**) Confirmation of anti-proliferative effects by the colony formation assay: SW480 and SW1116 cells formed much fewer and smaller colonies after treatment by 20 and 40 μM ATL for seven days (scale bar = 1 mm); (**e**) Quantification of colony formation assay results from three independent experiments: data were shown as average ± SD (*** *p <* 0.001 *vs.* vehicle control).

Recent studies found that ATL (Figure 1a) induced ROS accumulation and apoptosis in GBM glioblastoma [36], HepG2 hepatocarcinoma [37], RKO colon cancer [39], and Hela cervical cancer cells [38]. To examine the effects of ATL in more cancer cell types and to study the underlying mechanisms, we analyzed the cytotoxicity of ATL against the human SW480 and SW1116 colorectal cancer cells. SW480 and SW1116 cells, together with the non-cancer human BEAS-2B bronchial epithelial and human L-O2 liver cells, were treated by 20 μM ATL for 24 h and cell viability was measured by the MTT assay. The results showed that treatment with 20 μM ATL significantly inhibited the proliferation of the SW480 and SW1116 cancer cells, while the non-cancer BEAS-2B and L-O2 cells were not affected (Figure 1b). Detailed dose-response analyses showed that treatment by ATL for 24 h suppressed the proliferation of SW480 and SW1116 cancer cells with an *IC*_50_ of 21.63 μM (Figure 1c) and 18.14 μM, respectively.

Next, we used a colony formation assay to confirm the anti-proliferation activity of ATL. After treatment with 20 μM ATL for seven days, both SW480 and SW1116 cells formed much fewer and smaller colonies (Figure 1d,e), and higher concentration of ATL (40 μM) resulted in more complete inhibition. However, the growth of similarly-treated BEAS-2B and L-O2 non-cancer cells was not affected. Together, these results showed that ATL dose-dependently and specifically suppressed the growth of the SW480 and SW1116 colorectal cancer cells.

### 2.2. ATL Increased Reactive Oxygen Species (ROS) Levels in SW480 and SW1116 Colorectal Cancer Cells

**Figure 2:**
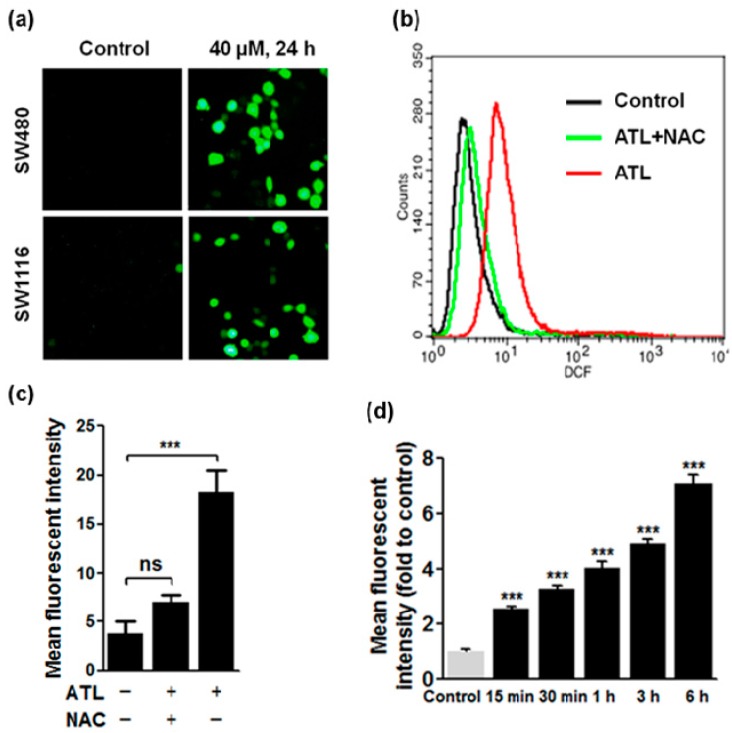
ATL significantly increased reactive oxygen species (ROS) levels in SW480 and SW1116 cancer cells. (**a**) Images of DCF fluorescence: treatment by 40 μM ATL for 24 h resulted in a robust increase in DCF fluorescent intensity in both SW480 and SW1116 cancer cells; (**b**) Flow cytometry analysis of total cellular ROS content: 6 h treatment by 40 μM ATL resulted in a four-fold increase in ROS levels, and pre-treatment with 5 mM *N*-Acetyl Cysteine (NAC) for 2 h blocked the ATL-induced ROS increase; (**c**) Quantification of flow cytometry data from three independent experiments (ns: no significant difference; *** *p* < 0.001 *vs.* vehicle control); (**d**) Time-dependent ROS accumulation: ROS levels rose significantly after treatment by 40 μM ATL for 15 min, and longer treatments resulted in higher ROS levels.

To check if ATL also induced ROS accumulation in SW480 and SW1116 colorectal cancer cells, we measured cellular total ROS levels by the fluorescent probe 2′,7′-dichlorofluoresceindiacetate (DCFH-DA). Treatment with 20 and 40 μM ATL for 24 h evoked a dramatic ROS increase in SW480 and SW1116 cancer but not the BEAS-2B and L-O2 non-cancer cells (Figure 2a). Quantification of ROS levels by flow cytometry revealed a four-fold increase in 40 μM ATL-treated cancer cells (Figure 2b,c). Pre-treatment with 5 mM *N*-Acetyl Cysteine (NAC) completely blocked the ATL-induced increase in DCF fluorescent intensity (Figure 2b,c), thus providing support to the specificity of the DCF signal. Significant increase in ROS levels was seen as early as 15 min after initiation of 40 μM ATL treatment (Figure 2d), and longer treatments resulted in stronger ROS increases (Figure 2d). These results suggest that treatment of SW480 and SW1116 colorectal cancer cells by ATL caused an immediate and robust increase in ROS levels.

### 2.3. ROS Overload Resulted in Extensive DNA Damage

**Figure 3:**
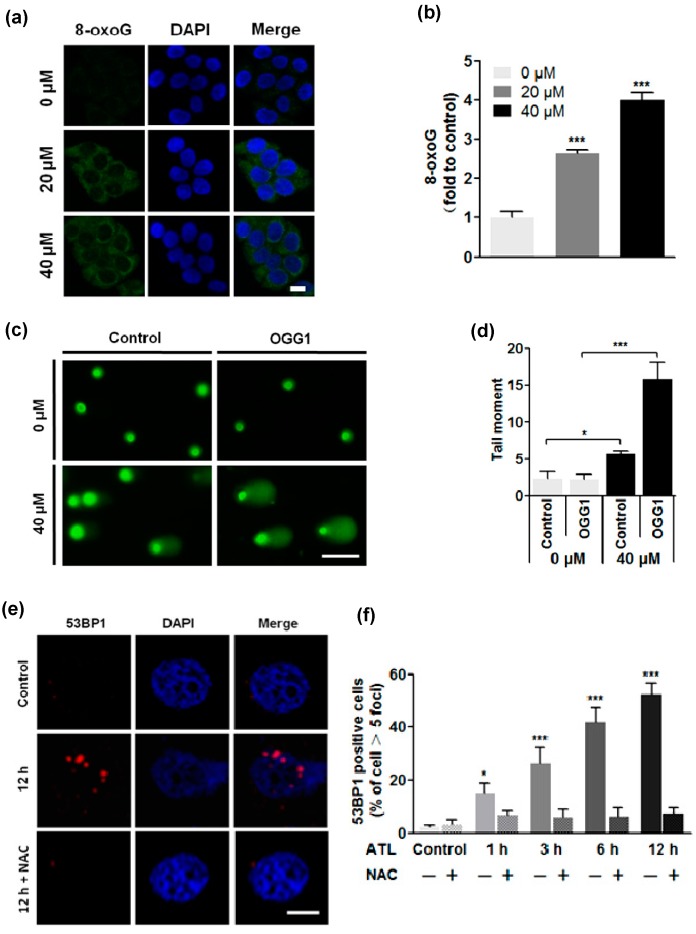
ROS overload resulted in extensive DNA damage. (**a**,**b**) Measurement of cellular 8-oxoG by Alexa 488-conjugated avidin: the level of cellular 8-oxoG was significantly increased in the colorectal cancer cells treated by 20 and 40 μM ATL for 24 h (scale bar = 20 μm) (*** *p* < 0.001 *vs.* vehicle control); (**c**,**d**) Comet assay: 24 h treatment with 40 μM ATL significantly increased the number of cells with comet tails and the size of the tails, and pre-incubation with OGG1 further promoted the increase (scale bar = 100 μm) (* *p* < 0.05, *** *p* < 0.001 *vs.* vehicle control); (**e**,**f**) Immunostaining of nuclear 53BP1: treatment with 20 μM ATL for 1 h prominently increased the number of 53BP1^+^ cells, and longer treatments resulted in more 53BP1^+^ cells; however, inclusion of 5 mM NAC in the treatment completely blocked the 53BP1 signal (scale bar = 5 μm) (* *p* < 0.05, *** *p* < 0.001 *vs.* vehicle control).

Elevated ROS levels may cause oxidative DNA damage that can trigger cell cycle arrest, premature cellular senescence or programmed cell death if the damages exceed cellular repair capacity. We reasoned that the ATL-induced ROS overload may have resulted in extensive oxidative DNA damage to cause the cytotoxicity of ATL. A significant target of ROS is the guanine nucleobase in both DNA and RNA molecules as well as the free nucleotides dGTP and GTP. Oxidation of guanine generates 8-oxoguanine (8-oxoG). To investigate if the elevated ROS levels in ATL-treated SW480 and SW1116 cells caused oxidative DNA damage, we first measured total cellular 8-oxoG by immunofluorescent staining with Alexa 488-conjugated avidin, which highly specifically binds to 8-oxoG [40]. The results revealed that treatment with 20 μM ATL for 24 h markedly increased cellular 8-oxoG level (Figure 3a,b). 40 μM ATL resulted in stronger staining, suggesting a dose–response relationship.

To further characterize the extent of DNA damage, we performed the comet assay (single-cell gel electrophoresis), which involves lysing agarose-embedded cells *in situ* and subjecting them to gel electrophoresis under alkaline conditions. Alkaline treatment converts all single-strand DNA breaks (SSB) into double-strand breaks (DSB). Undamaged DNA associates with nuclear proteins to form a highly organized structure in the nucleus and migrates as a whole, while the DNA with DSB migrates out of the nucleus, resulting in the appearance of a comet tail. The size and intensity of the comet tail is proportional to the number of DNA damages. Treatment with 40 μM ATL for 24 h significantly increased the number of cells with a comet tail and the size of the tails (Figure 3c,d), confirming the presence of a large number of DSB. Treatment with 5 mM NAC completely blocked the ATL-induced comet tails, indicating that the DNA damage was resulted from ROS. OGG1 is a glycosylase that specifically recognizes and removes 8-oxoG, leaving a DNA lesion which is converted into DSB under alkaline conditions. Pre-incubation with OGG1 further and significantly increased the number and size of the tails (Figure 3c,d), indicating the presence of a large number of 8-oxoG in the DNA.

To directly visualize and quantify cellular DSB, we performed immunofluorescent staining of 53BP1, which is an early marker of DSB [41]. The results showed that treatment by 20 μM ATL for 12 h dramatically increased the number of SW480 and SW1116 cancer cells with strongly stained nuclear 53BP1 foci (Figure 3e), while no significant change in 53BP1 signal was seen in similarly-treated non-cancer BEAS-2B and L-O2 cells. To characterize the timing of 53BP1 induction, the cancer cells were treated by 0, 20, and 40 μM ATL for 15, 30 min, 1, 3, 6, and 12 h. Increase in the number of 53BP1^+^ cells became significant after treatment by 20 μM ATL for 1 h (Figure 3f), and longer treatments (3, 6 and 12 h) resulted in more 53BP1^+^ cells (Figure 3f) as well as more cellular 53BP1 foci, all of which were blocked by treatment with 5 mM NAC (Figure 3e,f).

The results above indicate that ATL treatment caused an immediate and robust rise in ROS levels. Elevated ROS caused a marked increase in the level of cellular 8-oxoG, which includes those in DNA molecules (oxidative DNA damage marker) and the nucleotide pool (8-oxo-dGTP) (an important contributor of oxidative DNA damages) [42]. Oxidized nucleobases in DNA likely stimulated base excision repair (BER) to generate large numbers of SSB; and rapid increases in SSB saturated cellular repair capacity, resulting in numerous DSB [43]. Taken together, these results suggest that ATL treatment induced ROS overload, which subsequently caused massive oxidative DNA damage in the SW480 and SW1116 colorectal cancer cells.

### 2.4. ATL Induced Cell Cycle Arrest at G_1_

**Figure 4:**
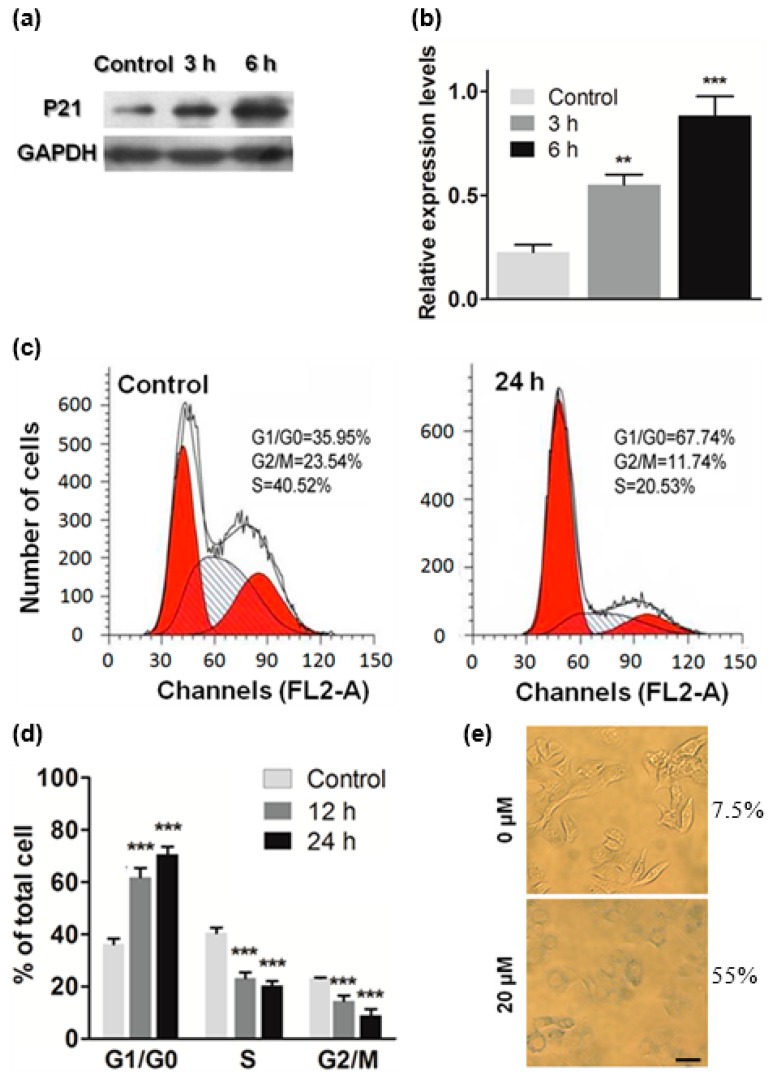
ATL treatment arrested the cell cycle at G_1_. (**a**,**b**) Western blot analysis of p21: treatment with 40 μM ATL for 3 h and longer increased the level of p21 in the colorectal cancer cells (** *p* < 0.01, *** *p* < 0.001 *vs.* vehicle control); (**c**,**d**) Examination of cell cycle by flow cytometry: the percentage of cells in G_1_ phase (left red area) was increased, while those in S (slash shaded area) and G_2_/M phases (right red area) were decreased by 40 μM ATL treatment (*** *p* < 0.001 *vs.* vehicle control) (note: the subG_1_ population was excluded from these analyses); (**e**) Detection of senescent cells: 20 μM ATL treatment for 48 h increased the percentage of cells with senescence-associated beta-galactosidase (SA-β-gal) activity (scale bar = 20 μm).

To investigate the consequences of the ATL-induced oxidative DNA damage, we analyzed the levels of some important proteins by Western blot. The results showed that treatment with 40 μM ATL for 3 h significantly increased the level of CDKN1B (p21) in the SW480 and SW1116 cancer cells (Figure 4a,b), and longer treatment (6 h) caused a bigger increase. As CDKN1B (p21) is a G_1_/S-CDK inhibitor and DNA synthesis suppresser, the result indicates that the ROS-induced oxidative DNA damage triggered DNA damage response (DDR) signaling to block the cell cycle.

Indeed, flow cytometry analysis revealed that treatment by 40 μM ATL for 24 h increased the number of cells in G_1_ phase (from 35.95% to 67.7%), at the same time, decreased the number of cells in both S phase (from 40.52% to 20.53%) and G_2_/M phases (from 23.54% to 11.74%) (Figure 4c,d). These results suggest that the oxidative DNA damages caused by ATL-induced ROS blocked the SW 480 and SW1116 cancer cells in G_1_ phase. Analysis of time-dependent inhibition showed that cell cycle arrest became significant 12 h after initiation of ATL treatment (Figure 4d).

Notably, treatment by 20 μM ATL for 48 h also induced significant cellular senescence as indicated by increase in senescence-associated beta-galactosidase (SA-β-gal) activity (Figure 4e).

### 2.5. ATL Induced Apoptotic Cancer Cell Death

**Figure 5:**
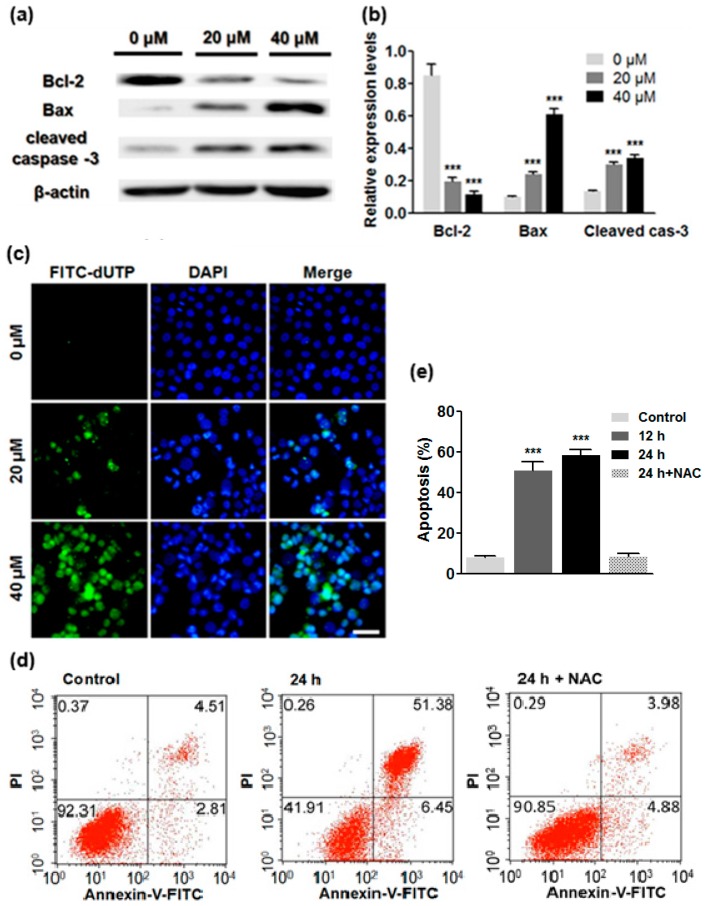
ATL treatment induced marked apoptosis. (**a**,**b**) Western blot analysis of apoptosis proteins: pro-apoptotic proteins Bax and active caspase-3 were significantly upregulated, while anti-apoptotic Bcl-2 was downregulated in SW480 and SW1116 cancer cells treated with 40 μM ATL for 24 h (*** *p* < 0.001 *vs.* vehicle control); (**c**) TUNEL assay: treatment by ATL for 24 h dose-dependently increased positively stained SW480 cells (scale bar = 100 μm); (**d**,**e**) Analysis of apoptosis by Annexin V-FITC and PI-double staining and flow cytometry: treatment of SW480 cancer cells by 20 μM ATL for 24 h caused marked apoptosis, which became significant 12 h after initiation of ATL treatment, and co-incubation with 5 mM NAC blocked the ATL-induced apoptosis (data were shown as average ± SD from three independent experiments, *** *p* < 0.001 *vs.* vehicle control).

In addition to CDKN1B (p21), Western blot results also showed that the pro-apoptotic proteins Bax and active caspase-3 were upregulated, while the anti-apoptotic Bcl-2 was downregulated in SW480 and SW1116 cancer cells treated by 20 and 40 μM ATL for 24 h (Figure 5a,b), suggesting the oxidative DNA damage caused by ROS also induced caspase-dependent apoptosis.

Based on these observations, we investigated some cellular apoptosis markers. First, fluorescent staining using the nuclear dye DAPI revealed that 24 h treatment with 20 and 40 μM ATL caused changes in nuclear morphology, which included an increase in hallmarks of apoptosis such as pyknosis and condensed chromatin (brighter nuclei); Second, the TUNEL assay was performed to directly visualize apoptotic cells, which revealed that 24 h treatment by 20 and 40 μM ATL dose-dependently increased positively stained cells (Figure 5c); Third, Annexin V-FITC staining and flow cytometry were used to follow changes in the number of apoptotic cells, which revealed that treatment by 20 μM ATL for 12 h and longer induced significant apoptosis in SW480 and SW1116 cancer cells (Figure 5d,e). After treatment of SW480 cells with 20 μM ATL for 12 and 24 h, the percentage of apoptotic cells increased from 7.32% to 54.17% and 57.83%, respectively (Figure 5d,e). Inclusion of 5 mM NAC inhibited ATL-induced apoptosis (Figure 5d,e), suggesting that ROS was the initial driver of apoptotic cell death.

**Figure 6:**
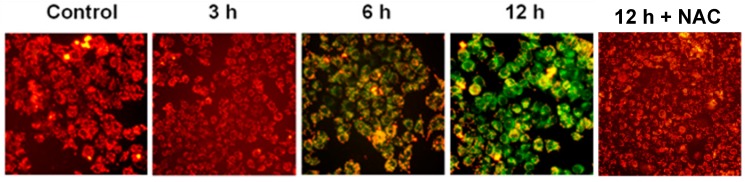
ATL treatment disrupted mitochondrial membrane potential. SW480 cells were treated with 20 μM ATL for 0.5, 1, 3, 6, 12, and 24 h or 20 μM ATL plus 5 mM NAC for 12 h, significant dissipation of mitochondrial membrane potential became evident after ATL treatment for 6 h and 5 mM NAC blocked the of mitochondrial membrane potential (scale bar = 50 μm).

Additionally, we examined the mitochondrial membrane potential by the JC-1 fluorescent dye. The resuls showed a prominent dissipation of mitochondrial membrane potential after treatment with 20 μM ATL for 6 h and longer (Figure 6), which was blocked by co-incubation with 5 mM NAC. Together, these results showed that the extensive oxidative DNA damage caused by elevated ROS activated the intrinsic apoptosis pathway.

## 3. Materials and Methods

### 3.1. Reagents and Cells

The human SW480 and SW1116 colorectal cancer cell lines and the immortalized human BEAS-2B bronchial epithelial cell line were from American Type Culture Collection (ATCC). The L-O2 human normal liver cell line was from KenGen Biotech (Nanjing, China). Alantolactone (ATL) was purchased from Yuanye Biotechnology (Shanghai, China). The purity was measured by HPLC and determined to be 99.6%. ATL was dissolved in DMSO and then diluted in cell culture medium to make working solutions.

### 3.2. MTT Assay

Cells were seeded in 96-well plates at 4 × 10^3^ cells per well and exposed to the indicated concentrations of ATL for 24 h. 20 μL of 5 mg/mL 3-(4,5-dimethylthiazol-2-yl)-2,5-diphenyl tetrazolium bromide (MTT) (Beyotime Biotechnology, Shanghai, China) was added to each well and the plate was kept at 37 °C for 4 h. After removing MTT, cells were incubated in 150 μL of DMSO on a plate shaker at 37 °C for 10 min. Absorbance at 490 nm was taken on a microplate absorbance reader (Bio-Rad Laboratories, Hercules, CA, USA). The GraphPad Prism software (San Diego, CA, USA) was used to process the data.

### 3.3. Colony Outgrowth Assay

Cells were treated with 0, 20, or 40 μM ATL for seven days and then fixed with ice-cold methanol for 20 min, followed by brief staining with crystal violet (0.5% in 25% methanol) (Sigma-Aldrich, St. Louis, MO, USA). After washing with PBS, the plate was dried overnight. After taking images, 70% ethanol was used to dissolve the violet crystals and the absorbance at 595 nm was taken on a microplate absorbance reader (Bio-Rad Laboratories).

### 3.4. Detection of Total Cellular ROS

Cells were treated with 40 μM ATL alone or together with 5 mM NAC for the indicated lengths of time, harvested, washed by PBS and incubated with 10 μM DCFH-DA diluted in DMEM for 30 min at 37 °C. After washing with DMEM for three times, DCF fluorescence intensity was analyzed by a fluorescence microplate reader (Infinite F200 Pro, TECAN, Männedorf, Switzerland). Alternatively, cells were collected by trypsinization. DCF fluorescence intensity was measured by the FACSCaliber flow cytometer (BD Biosciences, San Jose, CA, USA). Data from three independent experiments were quantified.

### 3.5. Examination of Cellular 8-Oxoguanine (8-oxoG)

Drug-treated cells were fixed in ice-cold methanol for 20 min, followed by PBS washing and incubation in Tris-buffered saline (TBS) plus with 0.1% Triton X-100 (Thermo Fisher Scientific, Waltham, MA, USA) for 15 min. After 1 h blocking in TBS with 0.1% Triton X-100 and 3% BSA, cells were stained by avidin-Alexa 488 (Thermo Fisher Scientific) for 1 h at 37 °C. PBS washed coverslips were then mounted in VECTASHIELD Medium with DAPI (Vector Laboratories, Burlingame, CA, USA).

### 3.6. Comet Assay

Drug-treated cells were suspended in 1.2% low-melting point agarose maintained at 37 °C. The agarose with the cells was then layered on a frosted slide from the OxiSelect Comet assay kit (Cell Biolabs, San Diego, CA, USA). The slide was submerged in pre-cooled lysis buffer (100 mM EDTA, 2.5 M NaCl, 10 mM Tris-HCl, 1% Triton X-100 and 10% DMSO, pH 10.0) and stored at 4 °C overnight. Slides were then washed twice with enzyme buffer (40 mM HEPES, 0.1 M KCl, 0.5 mM EDTA and 0.2 mg/mL BSA, pH 8.0) and then incubated in enzyme buffer or enzyme buffer with OGG1 (1.0 μg/mL) (Origen, Rockville, MD, USA) at 37 °C for 45 min. Slides were washed with enzyme buffer and denatured in pre-chilled alkaline buffer (300 mM NaOH, 1 mM EDTA) in an horizontal electrophoresis chamber (Bio-Rad Laboratories) for 30 min. Electrophoresis then proceeded at 20 V and 300 mA in the same buffer for 30 min. After incubation in cooled neutralizing buffer (250 mM Tris-HCl, pH 7.5) for 30 min, slides were immersed in cold 70% ethanol for 5 min and then allowed to air dry. Finally, cells were stained with Vista Green DNA dye at room temperature for 15 min in the dark. Images were acquired with an Olympus fluorescent microscope and quantified using the Comet Assay IV software (Perceptive Instruments, Edmunds, UK). Tail moment was defined as percentage of tail DNA x tail length, and more than 50 tailed cells were analyzed.

### 3.7. Immunostaining

After treatment by ATL or ATL together with 5 mM NAC, cells were fixed in 4% PFA for 15 min and blocked in 3% BSA with 0.1% Triton X-100 for 1 h at room temperature. PBS washed samples were then stained sequentially by rabbit anti-53BP1 (Bethyl Laboratories, Montgomery, TX, USA) and secondary anti-rabbit (Jackson ImmunoResearch Laboratories, West Grove, PA, USA) for 1 h at 37 °C. After PBS wash, the samples were sealed in Mounting Medium with DAPI (Vector Laboratories).

### 3.8. Western Blot

Cells were homogenated in RIPA (Santa Cruz Technology, Santa Cruz, CA, USA). After centrifugation at 12,000× *g* for 20 min at 4 °C, protein concentrations in the supernatants were measured by a BCA kit (Dingguo, Changchun, China). Denatured proteins were separated on 10%–12% SDS-PAGE gels and transferred to 0.2 μm nitrocellulose membrane. Blocking in TBS containing 5% non-fat milk and 0.5% BSA for 1 h at room temperature was followed by incubation with primary antibodies against Bcl-2 (Santa Cruz Biotechnology), Bax (Santa Cruz Biotechnology), cleaved caspase-3 (Abcam, Cambridge, UK), or p21 (Abcam) overnight at 4 °C, and then with a secondary antibody for 1 h at room temperature. Signals were revealed using an ECL kit (Bio-Rad Laboratories).

### 3.9. Cell Cycle Analysis

PBS washed cells were fixed in ice-cold 70% ethanol for 2 h at −20 °C, followed by incubation in working solution from a cell cycle detection kit (Bestbio, Shanghai, China) and filtration through a Filcon nylon mesh (BD Biosciences). Samples were analyzed by the FACSCalibur flow cytometer (BD Biosciences).

### 3.10. Detection of Cellular Senescence

Cellular senescence was revealed by detecting senescence-associated beta-galactosidase (SA-β-gal) activity using the SA-β-gal Staining kit (Beyotime Biotechnology). Cells were treated with 20 μM ATL for 48 h and fixed in 0.2% glutaraldehyde for 5 min at room temperature, followed by incubation in freshly-made staining solution at 37 °C overnight. After washing in PBS, photos were captured by an Olympus microscope. For quantification purpose, cells spanning three microscopic fields were manually counted.

### 3.11. Flow Cytometry Analysis of Apoptosis

After treatment with ATL or ATL together with 5 mM NAC for different periods of time, cells were collected, washed, and stained in working solution from an Annexin V-FITC/PI dual staining kit (Bestbio). Samples were analyzed by the FACSCalibur flow cytometer (BD Biosciences).

### 3.12. TUNEL Assay

Cells were exposed to 0, 20, or 40 μM ATL for 24 h, washed with PBS, and fixed in cold 4% PFA for 30 min, followed by incubation in 0.1% Triton X-100 in PBS for 2 min on ice. After washing twice in PBS, cells were incubated in working solutions from a One-Step TUNEL apoptosis assay kit (Beyotime Biotechnology) for 60 min at 37 °C. PBS washed coverslips were mounted in VECTASHIELD Medium with DAPI (Vector Laboratories).

### 3.13. Determination of Mitochondrial Membrane Potential

PBS washed cells were stained in the JC-1 fluorescent dye from a mitochondrial membrane potential assay kit (Beyotime Biotechnology) for 20 min and rinsed again in PBS. Slides were mounted in VECTASHIELD Medium (Vector Laboratories).

### 3.14. Statistical Analysis

Statistical analysis was performed by the GraphPad Prism software. Comparisons were calculated by one-way analysis of variance (ANOVA) and data were expressed as mean ± SD.

## 4. Conclusions

In this study, we investigated the anti-tumor effects and the underlying mechanisms of ATL, a natural sesquiterpene lactone, in human SW480 and SW1116 colorectal cancer cell lines. We found that ATL potently suppressed the growth and proliferation of colorectal cancer cells, while the growth of the non-cancer BEAS-2B and L-O2 cells was not affected. ATL treatment acutely increased cellular ROS levels (within 15 min of ATL treatment), and elevated ROS resulted in a dramatic increase in cellular levels of 8-oxoG, the number of DNA double-strand breaks and cells with bright 53BP1 foci, indicating induction of extensive oxidative DNA damages (within 1 h of ATL treatment). Consequently, the G_1_/S-CDK suppressor p21 and pro-apoptotic Bax and active caspase-3 were upregulated (within 3 h of ATL treatment), and dissipation of mitochondrial membrane potential was observed within 6 h of ATL treatment, which were followed by cell cycle arrest at G_1_ and activation of the intrinsic apoptosis pathway (within 12 h of ATL treatment). Suppression of DNA damage and apoptosis by NAC validates the critical role of ROS in ATL-induced cancer cell death. These studies provide further evidence showing that ATL has potent and selective anticancer activities that are related to induction of ROS overload and oxidative DNA damages. In addition, the results support promoting ROS overload as an important strategy for the development of new anticancer drugs.
